# Robust detection of event-related potentials in a user-voluntary short-term imagery task

**DOI:** 10.1371/journal.pone.0226236

**Published:** 2019-12-26

**Authors:** Min-Ho Lee, John Williamson, Young-Jin Kee, Siamac Fazli, Seong-Whan Lee

**Affiliations:** 1 Department of Brain and Cognitive Engineering, Korea University, Seoul, Korea; 2 Department of Computer Science, Nazarbayev University, Nur-Sultan, Kazakhstan; Kungliga Tekniska Hogskolan, SWEDEN

## Abstract

Event-related potentials (ERPs) represent neuronal activity in the brain elicited by external visual or auditory stimulation and are widely used in brain-computer interface (BCI) systems. The ERP responses are elicited a few milliseconds after attending to an oddball stimulus; target and non-target stimuli are repeatedly flashed, and the ERP trials are averaged over time in order to improve their decoding accuracy. To reduce this time-consuming process, previous studies have attempted to evoke stronger ERP responses by changing certain experimental parameters like color, size, or the use of a face image as a target symbol. Since these exogenous potentials can be naturally evoked by merely looking at a target symbol, the BCI system could generate unintended commands while subjects are gazing at one of the symbols in a non-intentional mental state. We approached this problem of unintended command generation by assuming that a greater effort by the user in a short-term imagery task would evoke a discriminative ERP response. Three tasks were defined: passive attention, counting, and pitch-imagery. Users were instructed to passively attend to a target symbol, or to perform a mental tally of the number of target presentations, or to perform the novel task of imagining a high-pitch tone when the target symbol was highlighted. The decoding accuracy were 71.4%, 83.5%, and 89.2% for passive attention, counting, and pitch-imagery, respectively, after the fourth averaging procedure. We found stronger deflections in the N500 component corresponding to the levels of mental effort (passive attention: -1.094 ±0.88 *μV*, counting: -2.226 ±0.97 *μV*, and pitch-imagery: -2.883 ±0.74 *μV*), which highly influenced the decoding accuracy. In addition, the rate of binary classification between passive attention and pitch-imagery tasks was 73.5%, which is an adequate classification rate that motivated us to propose a two-stage classification strategy wherein the target symbols are estimated in the first stage and the passive or active mental state is decoded in the second stage. In this study, we found that the ERP response and the decoding accuracy are highly influenced by the user’s voluntary mental tasks. This could lead to a useful approach in practical ERP systems in two respects. Firstly, the user-voluntary tasks can be easily utilized in many different types of BCI systems, and performance enhancement is less dependent on the manipulation of the system’s external, visual stimulus parameters. Secondly, we propose an ERP system that classifies the brain state as intended or unintended by considering the measurable differences between passively gazing and actively performing the pitch-imagery tasks in the EEG signal thus minimizing unintended commands to the BCI system.

## Introduction

For patients who suffer from neuromuscular diseases, such as locked-in-syndrome (LIS), paralysis, or spinal cord injuries, a brain-computer interface (BCI) [1] allows the user to control external devices by decoding his or her brain activity. Electroencephalography (EEG) has been widely used to record brain signals due to being non-invasive, low risk, and easy of use [2].

The P300 brain response is an exogenous potential that has a strong positive deflection in the EEG signal that is elicited approximately 300 ms after focusing on an odd-ball stimulus among the repetitive stimuli [3]. A typical application of a visual P300 evoked potential-based BCI includes a matrix of characters, commands, or other symbols [4, 5]. Typically, the target and non-target stimuli are randomly flashed while the EEG is monitored.

A P300 response is elicited only when the desired target symbol has flashed. Previous studies have utilized this effect to select the target stimuli. However, due to the low signal-to-noise ratio (SNR), the selection of a target from a single trial is difficult. Thus, the target symbol needs to flash several times in order to be detected. The epochs corresponding to each target and non-target trials are then averaged over the sequence in order to improve their accuracy. Consequently, several sequences of accumulative trial averages are required to select a single target stimulus. To reduce the required number of sequences and therefore minimize the consumption of time in the application, two basic strategies have been proposed to enhance the event-related potential (ERP) components.

One approach is to change the external characteristics of the target stimuli. Previous studies have reported that the exogenous potentials are highly influenced by the types of visual/auditory stimuli [6–10]. These studies among others have demonstrated that performance in a P300 experiment is influenced by various characteristics of the visual stimuli such as: the number of characters, the color of a symbol [7], and other experimental settings (e.g., matrix/symbol size or color, inter-stimulus interval) [11]. Several research groups have employed familiar face images [8, 9] or other novel types of motion-based stimuli [10] for presentation of the target symbol.

A second approach for enhancing the P300 component is to increase the user’s level of concentration on the target stimulus. Previous experiments compared the single-stimulus and odd-ball paradigms by evaluating the ERP response when users were instructed either to passively gaze or actively press a button [12–14] in response to visual and auditory stimuli, and it was found that active tasks generally elicited a more robust P300 response. To increase the user’s level of concentration on the target stimulus and thereby acquire a higher quality ERP component, most speller systems instruct users to focus on the target stimulus while conducting the simple task of silently counting the total number of target presentations [15, 16].

While most ERP-based BCI systems follow the first approach, it has some limitations as a practical application. In this study, our approach to enhance the ERP response followed the second strategy where user concentration was manipulated instead of the target symbols characteristics. Many past ERP-based BCI systems have required silent counting when the target stimulus is presented as a means to enhance the ERP response and prevent loss of attention during the experiment.

Other psychology based studies have used high/low pitch sounds as an auditory stimulus [17–19]. Recently, BCI systems have used auditory stimuli to control an external device, such as spellers [20, 21] and binary or multi-class interfaces [22–25]. In studies based on selective attention for a specific target, the user is passively listening for the target sound. This typically high-pitch sound is commonly experienced in daily life and is easy to imagine.

In this study, we asked subjects to employ a novel pitch-imagery task as a high-effort short-term imagery task that was to be done for every target presentation. Simply counting, or keeping a mental tally of the number of target presentations, was defined as a medium-effort short-term imagery task, and passively gazing at the target presentations without any additional task was considered low-effort. Therefore, we had two active tasks differing in levels of effort and one passive task. Our hypothesis is that the ERP responses would be highly influenced by the level of effort in user-voluntary tasks.

In the results, we observed significantly improved decoding accuracy and discriminative ERP patterns between the passive and imagery tasks. Specifically, the counting and and pitch-imagery task generated the strongest deflection in the N500 component compared to the passive task. The previous studies had reported this late ERP components; Falkenstein et al., [26] investigated the negative ERP components after 400 ms from the stimulus onset in Go/Nogo tasks and more earlier work suggests that this component is a correlate of error detection or inhibition [27]. Holcomb [28] reported that the target ERPs were measured in 200-525 ms (i.e., N400/P300) and also in 525-1100 ms as slow wave form. In the BCI study, those late ERP components have not been emphasized due to its less discriminant patterns compared to the N200 or P300 components. Our study investigated that this late ERP components are highly related to the user’s voluntary mental tasks and therefore it help not only to improve the decoding accuracy but also to potentially discriminate the user’s brain state being the passive (non-intended) or active mental (intended).

## Materials and methods

### Participants and data acquisition

This study was reviewed and approved by the Institutional Review Board at Korea University [1040548-KUIRB-16-159-A-2], and written informed consent was obtained from all participants before the experiments. A total of 14 healthy subjects (aged 25-33 years, 4 females) participated in this experiment. Five volunteers were naive to BCI tasks, and the other nine were familiar with some form of BCI task. All subjects reported being free of psychiatric or neurological disorders and had normal or corrected vision. Prior to the experiment, we explained the aims of our experiment, and the participants provided informed consent. The participants were seated in a comfortable chair at a distance of approximately 80 cm from the screen using a 19 inch LCD monitor (60 Hz refresh rate, 1280 × 1024 resolution).

EEG activity was acquired from a 32 channel (Fp1-2, F3-4, Fz, F7-8, FC5-6, FC1-2, T7-8, C3-4, Cz, CP1-2, CP5-6, TP9-10, P3-4, P7-8, Pz, PO9-10, O1-2, and Oz) ActiCap EEG amplifier (Brain Products, Munich, Germany) using Ag/AgCl electrodes according to the international 10-20 system, referenced on the nose with a forehead ground and an impedance of 10 k ohm or less. The sampling rate was 1000 Hz and a 60 Hz notch filter was used to remove the DC artifact. The EEG signal was band-pass filtered between 0.5 to 30 Hz using a 5^*th*^ order Butterworth filter.

### Experimental stimuli and paradigm

**Fig 1:**
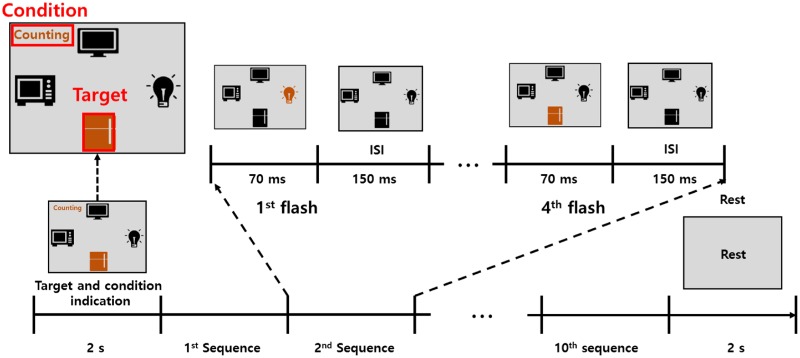
Visual stimulus for ERR experiment. Illustration of the experiment. Four visual symbols (microwave, refrigerator, bulb, and TV) were flashed in random order. The target symbol was flashed with an orange color.

The interface layout was designed with four target visual stimuli; TV, light bulb, refrigerator, and microwave. The stimuli were equally spaced on the screen in a configuration corresponding to the four directions (up, down, left, and right). The stimuli were black in color, and the background was gray (see Fig 1). The target stimuli randomly flashed with an orange color, and a single iteration of flashing in all directions was considered one sequence (4 flashes). A maximum of ten sequences were allotted where each set of stimuli flashed for 70 ms, followed by an inter-stimulus interval (ISI) of 150 ms. The presentation order was randomized, and no target stimuli were flashed consecutively in order to avoid a repetitive flash of the same target stimulus between the sequences. The paradigm code was developed with the Psychophysics Toolbox (http://psychtoolbox.com) and OpenBMI [29] in Matlab (MathWorks; MA, USA).

The three different tasks were defined and conveyed to the user before the sequences commenced. In the first condition (called passive ERP), the participants were instructed to gaze at the target stimuli in an idle state. In the second condition, participants were instructed to silently count each time the target stimulus was flashed. In the third condition, participants were given the novel instruction to conduct pitch imagery during target presentation. Before the experiment, a beep at a frequency of (8000 Hz) was presented to the participants for approximately 1 minute so that they could imagine the sound easily. Subjects remembered the tone and were instructed to imagine the pitch when the target stimuli appeared.

The paradigm was divided into two phases: the training phase and test phase. The three different conditions (i.e., passive, counting, and pitch-imagery) were conducted in both the training and test phases. To avoid performance bias, the order of each condition was counterbalanced and randomly assigned to the subjects. In the training and test phases, the participants were instructed to gaze at 20 symbols in each of the three different conditions.

Therefore, 200 target trials (20 symbols × 10 sequences × 1 target stimulus) and 600 non-target trials (20 symbols × 10 sequences × 3 non-target stimuli) were collected in a single session. All trials were assumed to be either targets or non-targets, regardless of the actual stimulus (e.g., microwave, TV, etc). When one specific symbol is cued to be the target stimulus (e.g., TV), all other symbols are considered to be equal non-targets (e.g., refrigerator, bulb and microwave). As a result, a binary classification task is obtained. By the end of the experiment, 400 target trials and 1200 non-target trials were collected in each of the passive ERP, counting, pitch-imagery paradigms. Note, that we collected three EEG datasets corresponding to each condition and the performance validations for the target vs. non-target or the passive vs. active state decoding were investigated based on those EEG datasets. The paradigm’s code was developed with the Psychophysics Toolbox (http://psychtoolbox.com) and OpenBMI [29] in Matlab (MathWorks; MA, USA). All the source-codes for this experiment were supported through GitHub (https://github.com/PatternRecognition/OpenBMI).

After completing the experiment, all subjects completed a simple questionnaire to evaluate their impressions of the passive, counting, and pitch imagery tasks. The questionnaire included the following three points of evaluation: 1. Difficulty: level of difficulty and discomfort (1 point: very easy, 5 points: very hard); 2. Concentration: level of concentration required during system use (1 point: very low, 5 points: very high); 3. Preference: level of likability for the three conditions (1 point: very low, 5 points: very high).

### Data analysis: Target decoder

Acquired EEG data were down-sampled to 100 Hz. The EEG data were segmented from -200 to 800 ms with regard to the stimulus onset and were baseline-corrected by subtracting the mean amplitudes in the -100 to 0 ms pre-stimulus interval from the epoch. The eight most discriminative intervals were extracted using a well-established method, the signed *r*-squared value [30], which statistically calculates the point-wise separability along the time series for target and non-target trials.

$$\begin{array}{c} r\left(t\right)=\frac{\sqrt{N_{1}\cdot N_{2}}}{N_{1}+N_{2}}\cdot \frac{{\bar{x}}_{i}^{1}-{\bar{x}}_{i}^{2}}{s_{i}} \end{array}$$ (1)

${\bar{x}}_{i}^{1}$ and ${\bar{x}}_{i}^{2}$ indicate the average of data samples at time point *i* corresponding to target (*mean*{*t*_*i*_|*target*}) and non-target (*mean*{*t*_*i*_|*non* − *target*}) trials, respectively. Where *t*_*i*_ represents the data samples at a certain time point *i* from the individual trials. *N*_1_ and *N*_2_ define the number of samples for target and non-target classes, respectively. *s*_*i*_ indicates the standard deviation of *t*_*i*_. The Cz, Pz, and Oz channels were used as reference channels to find discriminate time intervals. As a result, eight discriminant time intervals were manually selected from the reference channels and commonly used for all channels. The mean amplitude value in those 8 interval were separately calculated in the 32 individual channels and concatenated across channel dimension. These subject-dependent spatio-temporal feature vectors were therefore formed as ${\mathbb{R}}^{D\times 1}$ (*D* = 32 channels × 8 time windows). The regularized linear discriminant analysis (RLDA) [31] classifier was trained using these feature vectors of target and non-target trials in the training session.

$$\begin{array}{c} w={\mathrm{argmax}}_{w}\frac{w^{\text{T}}S_{B}w}{w^{\text{T}}S_{w}w} \end{array}$$ (2)

where **S**_**B**_ and **S**_**w**_ denote the between-class and within-class covariance matrices, respectively, and **w** is the hyper-plane for separation of the classes, which can be obtained by maximizing the Rayleigh coefficient.

$$\begin{array}{c} f\left(x\right)=w^{\text{T}}\cdot x+b \end{array}$$ (3)

where *b* is a bias term and *f*(**x**) is the classification output for the input feature vector **x**.

Let the individual ERP feature vectors $x_{ij}\in {\mathbb{R}}^{D\times 1}$, where *i* is the class index corresponding to the 4 stimuli flashes (i.e., microwave, refrigerator, TV, and bulb) at the *j* th sequence (*j* = 1, …, 10). $X\in {\mathbb{R}}^{D\times N}$ is the set of **x**, where *N* is the total number of trials (i.e., *i* × *j*).

$$\begin{array}{c} X=\{x_{11},x_{12},\ldots ,x_{ij}\} \end{array}$$ (4)

$v_{i}\in {\mathbb{R}}^{D\times 1}$ is the averaged ERP feature vector which is calculated by accumulatively averaging the current feature vector over the sequences *j* within the correct set of class index *i*.

$$\begin{array}{c} v_{i}=\frac{1}{n}\sum_{j=1}^{n}x_{ij} \end{array}$$ (5)

$$\begin{array}{c} V_{n}=\{v_{1},v_{2},v_{3},v_{4}\} \end{array}$$ (6)

where $V_{n}\in {\mathbb{R}}^{D\times i}$ indicates a set of averaged ERP features for the four classes (*i*) at the certain sequence number *n*. For instance, **v**_3_(*n* = 4) indicates the ERP feature vector for the 3rd class (i.e., TV) that was the accumatively averaged result from **x**_3,1_, **x**_3,2_, **x**_3,3_, and **x**_3,4_.

The classification score *f*(**V**_**n**_) is calculated from the input feature vectors **v**_*i*_(*i* = 1, …, 4), and target class, which has the highest classification score, is then estimated from this four class index *i*.

$$\begin{array}{c} {\hat{i}}_{n}={\mathrm{argmax}}_{i}\left(f\left(V_{n}\right)\right) \end{array}$$ (7)

where *î*_*n*_ is the estimated index for the target class after a certain sequence *n*. Note that the target decoder is generated based on the binary classifier for classification of target or non-target classes. Specifically, *i* is the index of the four classes, and **v**_*i*_ has the individual features corresponding to the class index. The target decoder selects the class index *î* which has the maximum classifier output from the four individual features (i.e., *f*(**v**_*i*_)). The system performance is calculated for every sequence individually.

The target decoder was able to predict a target symbol from the four visual stimuli (chance level at 25%). The performance of the three separate paradigms were investigated by the same procedure as described in this section. The collected data during the training phase were used to calculate classifier parameters, and the data in the test phase were used for performance validation.

### Data analysis: Active decoder

To classify active and passive mental states, only the target trials from the three conditions were selected to build the classifiers. Two binary classifiers were created to classify passive gazing vs. either counting or pitch-imagery. In the case of the active decoder for classification of passive vs. pitch-imagery, sets of target trials *X*_*pa*_ and *X*_*pi*_ were extracted from the dataset and fed into the LDA classifier. The performance validation for passive gazing vs. the counting or pitch-imagery task was also calculated sequentially (by averaging the currently acquired ERP data), described in the previous section. The active decoder was able to predict a user’s mental state as to whether he or she was gazing at a target symbol in passive or intentional state with a chance level at 50%.

### Data analysis: Two-stage classification

In this section, our two-stage classification strategy to predict a target symbol in addition to the intentional mental state is described. The classification strategy was basically designed by combining two individual classifiers: the target decoder (*f*_**T**_) and the active decoder (*f*_**A**_), described in the previous sections. Given the averaged ERP feature vector **v**_*i*(*i*=1,…,4)_ acquired ERP trial **v**_*i*(*i*=1,…,4)_, the classification scores of all four symbols were calculated based on the target decoder and the symbol with the highest score was estimated as the target (i.e., *î* = argmax_**i**_ *f*_**T**_(**v**_*i*_)).

In the second step, the given ERP feature vector **v**_*î*_ that was estimated as a target symbol was fed into the active decoder *f*_**A**_ to determine whether a user was gazing at a target symbol with or without any intention of command.

$$\begin{array}{c} y=\text{sgn}(f_{A}(v_{\hat{i}})) \end{array}$$ (8)

where the *y* (*y* ∈ [−1, 1]) is binary decision corresponding to the user’s current mental states, i.e. the active state (*y* = −1) or the passive state (*y* = 1).

## Results

### Target vs. non-target decoding accuracy

**Fig 2:**
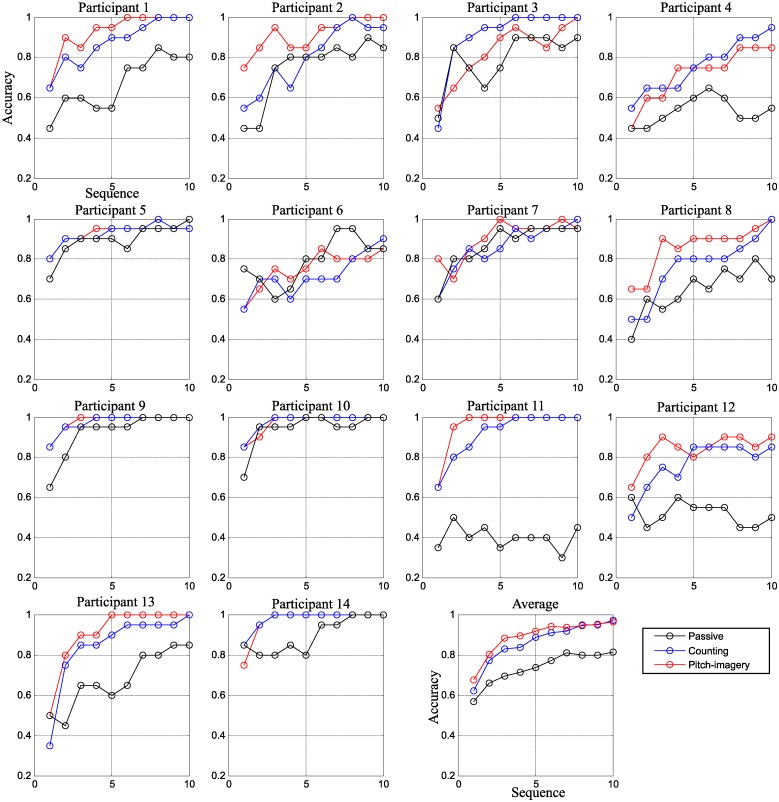
Sequential decoding accuracy in three conditions. The decoding accuracy for individual subjects and their average. The performance (y-axis) was calculated from one to a maximum of ten sequences (x-axis).

Fig 2 indicates the decoding accuracy for target and non-target discrimination in the three conditions. The decoding accuracy were evaluated from sequences one to ten (x-axis) for individual subjects along with the average performances. The accuracy of the passive, counting, and pitch imagery tasks were indicated by black, blue, and red lines, respectively. The results revealed that the active tasks (i.e., counting and pitch-imagery) achieved higher accuracy than the passive task. Specifically, the average accuracy were 71.4%, 83.5%, and 89.2% after sequence four, and 81.4%, 97.1% and 96.4% after sequence ten for the passive attention, counting, and pitch-imagery tasks, respectively.

We perform a one-way, Bonferroni corrected ANOVA for the three conditions (passive, counting, and pitch-imagery) with the null hypothesis of equal means to the decoding accuracy. Before the statistical test, we validated the accuracy distributions for each sequence in all conditions with the Jarque-Bera test for normality. Sequences were normally distributed except the 10th sequence which was only marginally skewed with a p-value of 0.0402. The results of the ANOVA showed significant differences at sequences 5, 6, 8 and 10 (*p* < 0.05) for counting vs. passive attention. The pitch-imagery task had significantly higher accuracy than the passive condition for sequences 3 to 10 (*p* < 0.05). For the performance comparison between the two active tasks, the pitch-imagery condition achieved a significantly higher decoding accuracy as compared the counting task at sequence four (*p* < 0.05).

**Fig 3:**
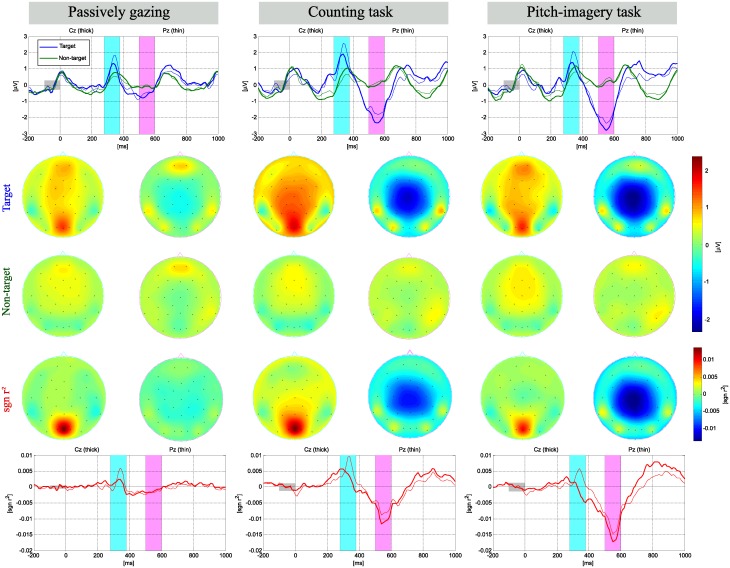
Grand averaged ERP and signed *r*^2^ in individual tasks. Grand averaged visualization of the ERP and signed *r*^2^ at the Cz and Pz electrodes with scalp topographies corresponding to specific time intervals (blue bar: 280-380 ms, pink bar: 500-600 ms).

Fig 3 shows grand averaged ERP responses for target and non-target trials in the three different conditions. The entire data sets of training and test sessions were combined for those sets where the same experimental protocol was used. The typical shape of the ERP responses with regards to the N200 and P300 components for the target and non-target visual stimuli are seen as has been reported in [12, 13, 32]. The ERP responses in the interval of the P300 and N500 components are indicated by sky-blue (280-380 ms) and pink bars (500-600 ms), respectively, as these intervals demonstrated the most discriminative patterns in the three different conditions.

The mean of peak amplitude for target and non-target trials was calculated in the intervals of N200 (180-230 ms), P300 (290-390 ms), and N500 (500-600 ms) at the Oz and Cz electrodes. The peak amplitude at the Cz electrode, N200 was -0.557 ±0.63*μ*V, -0.422 ±0.89*μ*V, and -0.855 ±0.90*μ*V; the P300 was 1.187 ±1.02*μ*V, 3.029 ±1.48*μ*V, and 3.208 ±1.70*μ*V; and the N500 was -1.094 ±0.88*μ*V, -2.226 ±0.97*μ*V, and -2.883 ±1.34*μ*V for the passive attention, counting, and pitch-imagery tasks, respectively. The peak amplitudes in the Oz electrode showed relatively low discriminate value between each task (see the details in Table 1).

**Table 1:** The mean of peak amplitudes for the three ERP components (N200, P300, and N500) at Oz and Cz electrodes.

Ch.		Oz			Cz		
Components		N200	P300	N500	N200	P300	N500
Pa.	Target	-2.217(±1.51)	2.628(±1.33)	-1.107(±0.98)	-0.557(±0.63)	1.817(±1.02)	-1.094(±0.88)
	N-target	-0.427(±0.45)	0.602(±0.46)	-0.711(±0.52)	-0.911(±0.47)	1.133(±0.49)	-0.663(±0.48)
Co.	Target	-2.213(±1.26)	4.009(±1.39)	-1.147(±1.08)	-0.422(±0.89)	3.029(±1.48)	-2.226(±0.97)
	N-target	-0.553(±0.34)	0.566(±0.52)	-0.641(±0.46)	-1.277(±0.64)	1.268(±0.60)	-0.532(±0.69)
Pi.	Target	-2.351(±1.18)	3.646(±1.14)	-1.088(±1.13)	-0.855(±0.90)	2.308(±1.70)	-2.883(±1.36)
	N-target	-0.493(±0.34)	0.671(±0.34)	-0.673(±0.47)	-1.284(±0.73)	1.384(±0.89)	-0.541(±0.74)

**Fig 4:**
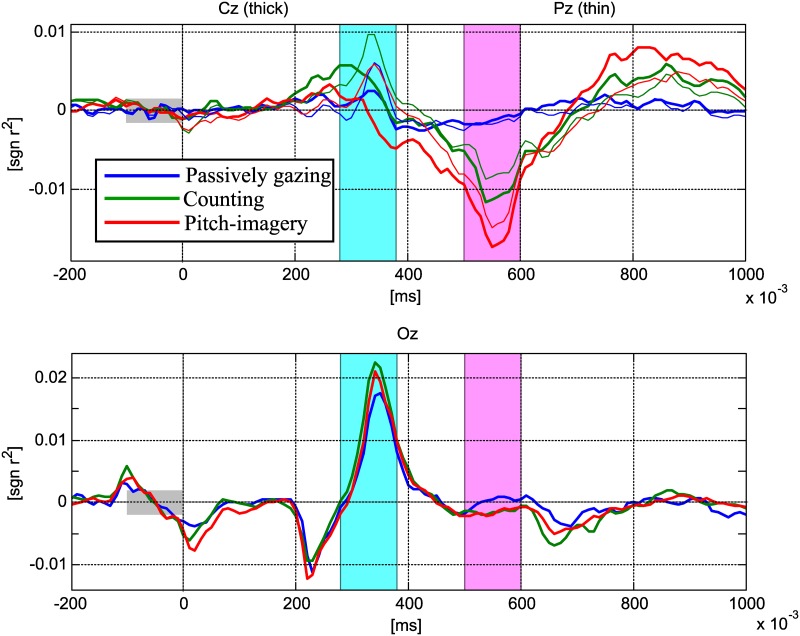
The signed *r*^2^ value for target and non-target trials. Grand averaged signed *r*^2^ values for target and non-target trials. Upper plot indicates signed *r*^2^ at channel Cz and Pz for observing the P300 and N500 components, while bottom plot indicates signed *r*^2^ at channel Oz for observing the N200 and P300 components (blue colored line: passive, green colored line: counting, red colored line: pitch imagery).

Fig 4 indicates the signed *r*^2^ [33] value for the target vs. non-target trials for the passive gazing (blue line), counting (green line), and pitch-imagery (red line) tasks. The P300 (240-280 ms) and N500 (500-600 ms) intervals are again indicated in sky-blue and pink bars, respectively. The upper plot indicates the signed *r*^2^ value at the Cz (thick line) and Pz (thin line) electrodes for the comparison of the P300 and N500 components, while the bottom plot indicates the *r*^2^ value at the Oz electrode for the comparison of the N200 and P300 components. In the upper plot (ERP responses at the Cz electrode), the counting task achieved the highest positive *r*^2^ value in the P300 interval, followed by the pitch-imagery and passive attention tasks. On the other hand, the pitch-imagery task achieved the highest negative *r*^2^ value in the N500 interval, followed by the counting and passive attention tasks.

The N200 and P300 components in the Oz and Cz electrodes were commonly observed in the three different conditions as reported in many previous studies [14, 34, 35]. Furthermore, we found that the N500 components at the Cz electrode achieved a highly discriminative *r*^2^ value for each task. Specifically, the two active tasks (i.e., counting and pitch-imagery) achieved high *r*^2^ values for discriminating the target and non-target trials, while the passive attention task exhibited a relatively non-discriminative pattern in the 500-600 ms interval.

The *r*^2^ value at the Oz electrode (bottom plot) indicated that all *r*^2^ values over the time course were relatively similar ERP responses in all conditions; however, the counting and pitch-imagery tasks achieved higher *r*^2^ values than the passive condition in the 240 ms-380 ms interval (i.e., P300).

Table 2 presents the target vs. non-target decoding accuracy using ERP features, extracted from three individual time intervals; 150-250 ms (N200), 280-380 ms (P300), and 500-600 ms (N500) after sequence three. These intervals were chosen to include neurophysiological components, such as N200 and P300 that are widely known in the literature. In addition a time interval that included the N500 component that was evoked by the mental task (e.g., pitch-imagery) was also examined. For the designated intervals (i.e., 150-250 ms, 280-380 ms, and 500-600 ms), mean amplitude feature was calculated over the 32 channels. Therefore, the feature vectors were formed with 32 dimensions (i.e., 1 × 32). For the 0-800 ms interval, on the contrary, the mean amplitude features in those three intervals were simply concatenated (e.g., 1 × 3 × 32).

**Table 2:** The decoding accuracy in the specific time intervals (150-220 ms, 280-380 ms, and 500-600 ms) for the passive attention (pa.), counting (co.) and pitch-imagery (pi.) tasks. ** indicate *p* < 0.01.

Sub.	150-250ms			280-380ms			500-600ms			0-800ms		
	Pa.	Co.	Pi.	Pa.	Co.	Pi.	Pa.	Co.	Pi.	Pa.	Co.	Pi.
1	60	70	30	50	65	60	25	25	50	60	75	85
2	30	45	30	50	40	75	25	45	65	75	75	95
3	65	75	45	55	70	55	20	40	65	75	90	75
4	20	20	40	40	40	40	35	50	55	50	65	60
5	45	45	60	75	70	80	35	55	60	90	90	90
6	65	35	40	45	30	60	25	30	30	60	70	75
7	65	40	35	30	80	60	30	70	65	80	85	85
8	30	20	35	30	40	50	30	45	25	55	70	90
9	55	65	60	85	70	65	45	35	60	95	95	100
10	45	65	65	80	80	85	40	45	75	95	100	100
11	30	30	65	20	60	45	35	40	65	40	85	100
12	35	35	35	30	60	50	40	45	40	50	75	90
13	65	50	50	40	70	70	25	50	50	65	85	90
14	35	80	70	80	95	90	25	85	65	80	100	100
**Mean (std)**	46.1 (15.6)	48.2 (19.1)	47.1 (13.7)	50.7 (20.6)	62.1 (17.8)	63.2 (14.5)	31.1 (7.1)	47.1 (14.8)	55.0** (14.0)	69.3 (16.9)	82.9 (10.9)	88.2 (11.2)

The results indicate that the P300 interval achieved decoding accuracy of 50.7%, 62.1%, and 63.2%, and N500 interval achieved decoding accuracy of 31.1%, 47.1%, and 55.0% for the passive, counting, and pitch imagery conditions, respectively. We apply a one-way, Bonferroni corrected ANOVA test with the hypothesis of equal means to the decoding accuracy of the time intervals of 150-250 ms, 280-380 ms and 500-600 ms as well as 0-800 ms, resulting in *n*(*n* − 1)/2 = 66 individual tests (*p*-values). For the interval of 500-600 ms, we observed significant differences only for passive vs. pitch imagery conditions (*p* < 0.01).

**Fig 5:**
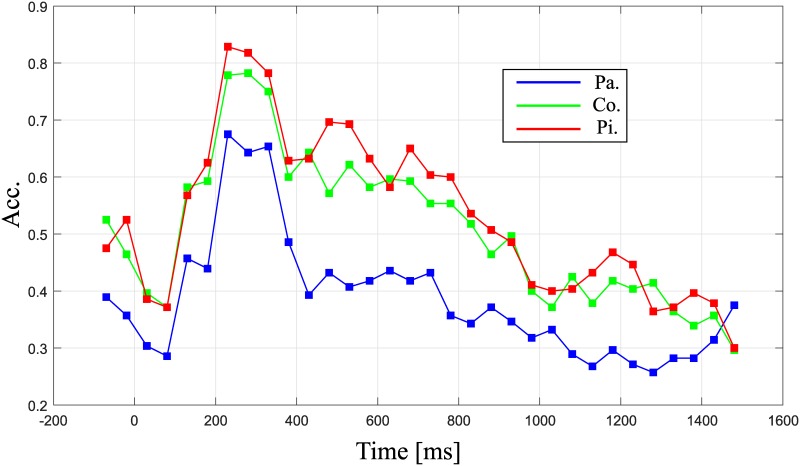
The grand average decoding accuracy in the temporal domain. The decoding accuracy for target and non-target trials in the temporal domain using a sliding window of 50 ms (buffer size of 100 ms). The time intervals of 190-310 ms and 440-560 ms patched in gray are the intervals of importance in the ERP components (i.e., N200, P300, and N500) and exhibited significant difference in their performance.

Additionally, the decoding accuracy over the time courses were investigated by short time segment analysis (a sliding window of 50 ms with buffer size of 100 ms). The most discriminant information was found in the two intervals of 190-310 ms and 440-460 ms, representing the N200/P300 and N500 components, respectively. Furthermore, the performance difference between the pitch-imagery and counting task mostly appeared in the N500 component (see Fig 5).

### Active task vs passive tasks accuracy

**Fig 6:**
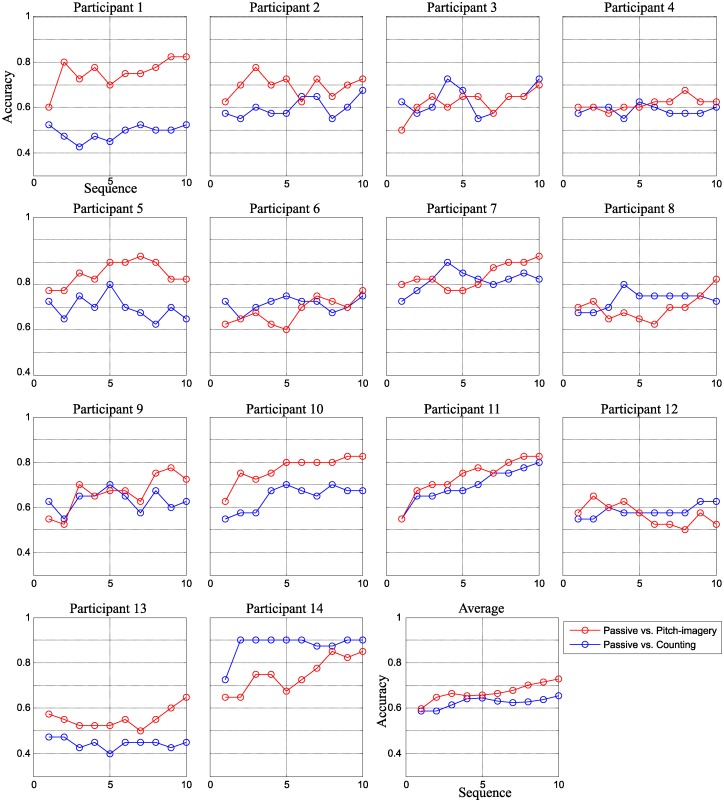
The sequential decoding accuracy for passive vs. active decoder. The sequential decoding accuracy of all individual subjects for passive vs. pitch-imagery decoding (red line) and passive vs. counting decoding (blue line).

Fig 6 indicates the decoding accuracy of the passive task vs. the active tasks for individual subjects along with their averages. The decoding accuracy (y-axis) were calculated with respect to the number of sequences (x-axis). The number of sequences varied from one to ten. The decoding accuracy at sequence ten were 66.2% for counting vs. passive attention and 73.5% for pitch-imagery task vs. passive attention. The high-pitch imagery task significantly outperformed the counting task for discriminating the active vs. passive mental state at sequences eight, nine, and ten (*p* < 0.05).

Therefore, the active decoder was generated based on the binary classes of pitch-imagery and passive attention. The decoding accuracy of two-stage classification strategy was 71.6%. Here, we define: true positive (TP) as successful decoding of the target symbol as well as the user’s active mental state, true negative (TN) as successful decoding of the user’s passive mental state, false positive (FP) as when the decoder failed to estimate either the target symbol or user’s active mental state or both, false negative (FN) was when the decoder failed to estimate the user’s passive mental state. Please note that the passive mental stage in two-stage classification does not consider whether the target decoder successfully estimate the target symbol or not. From this cases, true positive rate (TPR), false positive rate (FPR) were 0.716 and 0.245, respectively.

**Fig 7:**
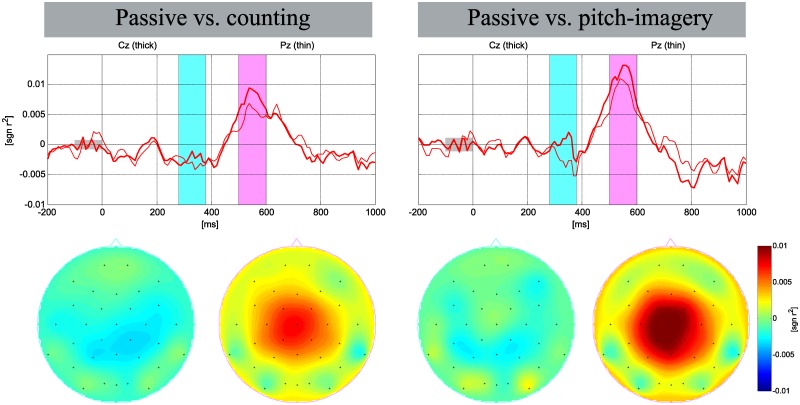
The signed *r*^2^ values for active decoding. The signed *r*^2^ at the Cz (thick red line) and Pz (thin red line) electrodes for the passive vs. active conditions. The blue and pink bars represent the P300 and N500 components, respectively.

Fig 7 presents the signed *r*^2^ values for the passive task vs. active tasks. The signed *r*^2^ value indicates that the N200 (180-220ms) and P300 (280-380ms) components did not affect the decoding performance; however, the N500 (500-600ms) component achieved a markedly higher *r*^2^ value for decoding the passive vs. active conditions. Furthermore, the pitch-imagery task achieved a higher *r*^2^ value in the N500 interval than did the counting task.

### Questionnaire

**Fig 8:**
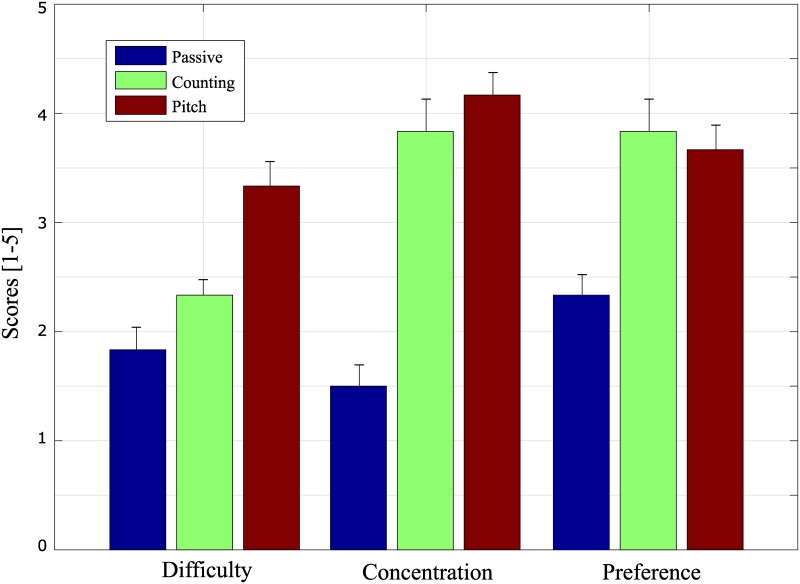
The mean rating scores of the questionnaire for all participants. Difficulty (1 point: very easy, 5 points: very hard), concentration (1 point: very low, 5 points: very high), and preference (1 point: very low, 5 points: very high).

Fig 8 illustrates the mean scores corresponding to the difficulty, required concentration, and preference of the tasks. The participants reported that the counting and pitch imagery tasks were relatively difficult compared to the passive task. All 14 users reported that the active tasks required higher concentration than the passive tasks. Additionally, the active tasks were preferred over the passive.

## Discussion

### User voluntary pitch-imagery task in BCI

In the study of BCI, much research has been undertaken to increase system performance by boosting the ERP amplitude. Various factors influence the ERP components, but the majority of previous studies have focused on manipulation of external experimental parameters, such as the size/shape/color/face of the stimuli [7–11]. Only a few BCI studies have investigated the internal user side factors [12, 13, 15]. One example, Markus et al. [15] investigated whether the neural activity is specific to the counting task, or if it can also be evoked by other tasks that direct the attention to certain stimuli. The results indicated that the neural activity is not strictly task-specific, and can be generalized over tasks and is presumably a result of attention allocation or of the augmented workload.

The counting task is very simple, and all of the users could easily perform the task without psychological difficulty. On the other hand, the pitch-imagery task required more mental effort; the users typically have little to no experience performing such a task. Regardless, a beep sound is a familiar sound from daily life, and all users could easily perform the pitch-imagery task after a 5 minute practice session. Previous studies have presented a high-pitch sound as an auditory stimulus [17–19], which motivated us to propose a user voluntary pitch-imagery task. In our experimental results, the majority of the users (13 of 14) successfully generated the high amplitude ERP component in the N500 interval.

In this study, the system performance significantly improved through the user’s own effort based on task demand without any external help. This demonstrates that the system could prove to be practical and usable as the BCI systems can be designed simply without any additional setup. In this respect, the pitch-imagery task could be substituted in place of previous boosting methods or used in conjunction.

### Influence of task level on the BCI performance

In this study, we explored the change in the ERP response with regard to the level of mental task, and found that it contributed to robust decoding of the neural activity for target and non-target trials. We observed the ERP patterns obtained in response to different mental tasks (i.e., passive gazing, counting, and pitch-imagery). Fig 4 indicates the signed *r*^2^ values for the target vs. non-target trials at the Oz, Cz, and Pz electrodes. The signed *r*^2^ value for the Oz electrode (bottom plot) indicated typical ERP responses for the N200 and P300 components with no prominent difference between the three conditions because the occipital cortex directly reflects the visual stimuli from the retina [14, 34, 36].

On the contrary, the ERP patterns in central cortex (i.e., Cz, and Pz electrodes) were highly discriminative between each condition. First of all, the three tasks commonly generated a discriminant P300 component. This result indicates and confirms the well-known result that the P300 component in the central cortex area is quite robust and is able to decode target and non-target trials [36]. However, the active tasks evoked more discriminative P300 components than the passive task, which significantly influenced the decoding accuracy (see Tables 1 and 2). There was no significant difference in performance between the active tasks although the counting task generated a slightly stronger P300 component compared to the pitch-imagery task. However, the decoding accuracy was slightly higher in the pitch-imagery task. The performance distinction between the active tasks was mainly determined by the N500 component (see Fig 5).

Significantly discriminant N500 components in the averaged ERP responses were found in the central cortex area (the Cz, and Pz electrodes). It is clear that the N500 components are evoked by the mental effort of the active tasks. Passive gazing generated a relatively weak deflection of amplitude while the counting and pitch-imagery tasks generated markedly stronger deflections in the N500 component (see Fig 3). Moreover, the pitch-imagery task achieved the strongest *r*^2^ value in the N500 interval for discriminating target and non-target trials (see Table 2 and Fig 5) compared to the passive and counting tasks. Therefore, the N500 is highly related to the task-relevant component and is the main reason for showing the best performance in pitch-imagery task (see Fig 5).

Table 2 presents the decoding accuracy of the three important ERP components. The purpose of this table is to show how each component (N200, P300, and N500) is evoked by the different mental tasks. The decoding accuracy presented are at the 3rd sequence, however, the accuracy of the P300 at the 10th sequence are 63.3% (passive), 80.7% (counting), 82.1% (pitch-imagery) and for the N500 36.7%, 56.4%, 66.4%. In fact, the most discriminant interval was the 280-380ms (i.e., P300 component). Please importantly note that the N500 component in active tasks is beneficial in improving classification performance beyond the baseline performance but it alone does not provide a robust or reliable signal for classification accuracy.

Previous ERP studies have mainly focused on the N200 or P300 components while the N500 component has not been sufficiently investigated. Only a few studies mentioned a negative ERP component around 400-500 ms and they demonstrated varied amplitude differences in that interval due to stimulus type [24, 37, 38], level of concentration [39], and other factors [40, 41].

Halder et al. [24] proposed an auditory BCI based on a three-stimulus paradigm with difference in loudness, pitch, or direction. The best performance was achieved with targets differing in pitch. They found significant differences in the intervals of 300 ms and 550 ms for target and non-target ERP data. Park et al. [37] investigated the difference of P300 and N500 components by the deviant tones in the auditory oddball task. The study of [39] investigated the influence of odorant concentration on the olfactory ERP system. The different concentrations of vanillin-saturated air were splayed to the user’s right nostril while the visual stimuli presenting. They reported discriminative ERP responses around 500 ms by the level of odorant concentration. All of the studies mentioned directly above investigated the ERP responses in regard to the external experimental factors. However, difficulty exists in applying these boosting methods on real-world BCI applications due to some strict limitations. For instance, such auditory or olfactory settings are not suitable for outside applications due to the deterioration of stimuli. Additionally, certain types of visual stimuli [16, 42–44] are also inappropriate if required to be used by LED-type or a small sized embedded monitors.

The experiment results in our study prove that a stronger ERP responses can be derived not only from the experimental stimuli but also from the user’s internal capacities. Smaller size and less visually effective stimuli could greatly reduce system complexity or lead to greater visual fatigue [45] with a loss in decoding accuracy. However, the boost from higher-effort user-voluntary active tasks could supplement this deficit by maintaining the class discriminant power in the ERP responses.

The mental effort required for target selection involves maintaining a high level of concentration during the experiment and is an important factor for stable BCI performance. A previous study reported that perceived mental effort reflects changes in arousal during attention-based tasks [46]. It was found that perceived mental effort increased when the demands of the task increased; the ERP amplitude and the latency were changed according to the different attentional tasks. To prevent a loss of attention during the experiment, most of the past ERP studies on the oddball paradigms have basically instructed participants to perform a counting task [8, 9, 15, 47].

The task is not compulsory; users check whether they correctly counted the number of stimuli by themselves. The counting task has not been considered as an additional user-voluntary task in furtherance of improving the decoding accuracy and has been used without sufficient investigation into the actual effect of the counting task on the ERP responses. In this study, we definitely consider the counting and pitch-imagery as an additional user-voluntary mental task. The clear effects of those active tasks on ERP responses and their influence on decoding accuracies are reported here as another step in increased understanding of how task demands effect ERP responses for BCI decoding accuracy.

The prolonged use of a ERP-based BCI systems may unintentionally reduce performance for many reasons. Primarily, the user becomes accustomed to the repetitive visual or audio stimulus. The passive gazing state, in particular, would be susceptible to reduced system performance as time progresses [48, 49].

**Fig 9:**
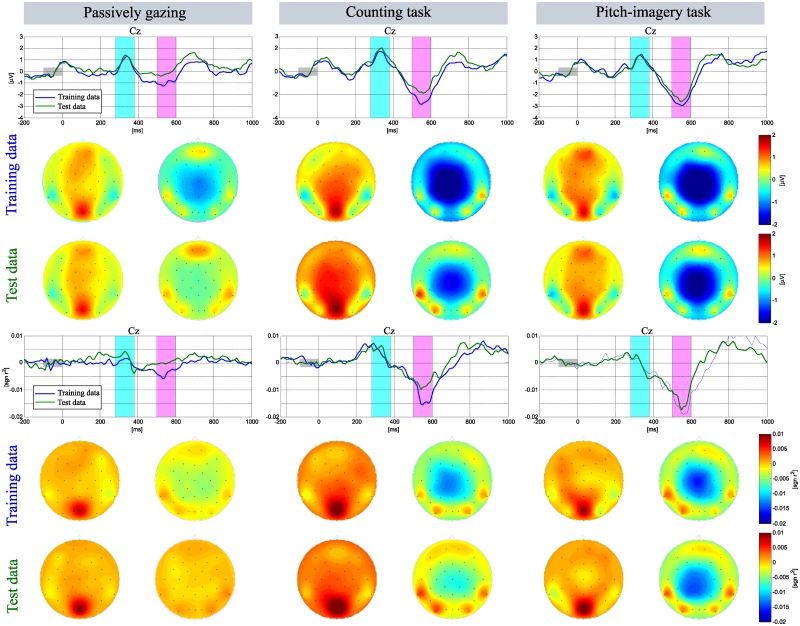
Changes in the ERP responses between the training and test sessions. Grand averaged visualization of ERP and signed *r*^2^ with the scalp topographies.

Fig 9 indicates the ERP responses for the training and test sessions. The upper plot indicates the ERP responses of the Cz electrode for the training data (blue line) and test data (green line). We observed interesting ERP responses for the training and test sessions; the N200 and P300 components were not different between the sessions, however, the amplitude of the N500 component decreased during the test session.

More importantly, the passive gazing and counting tasks induced a strong deflection in the N500 component and a decrease in the signed *r*^2^ value for target vs. non-target (see bottom plot in Fig 9), while the pitch-imagery task relatively preserved the N500 amplitude and the signed *r*^2^ value. Our results suggest that high-effort voluntary mental tasks (such as pitch-imagery) would not only improve decoding accuracy but also maintain performance in prolonged usage of the BCI system.

### Practical implications to the ERP applications

In our study, we reported discriminative ERP patterns between passive and active attention, which inspired us to classify the intentional or unintentional mental state. Specifically, the N200 and P300 components are commonly evoked by a target stimulus, however, the N500 components exhibited highly discriminative patterns not only between the target and non-target trials but also between the passive and active mental states. The N500 component is therefore potentially usable for classifying an active or passive mental state. The N500 component obtained a mean decoding accuracy of 73.5% for classifying the passive gazing vs. pitch-imagery task, which is acceptable for brain-computer communication, [50] and this result motivated us to develop more practical and usable ERP applications.

Previous ERP-based applications have been developed using an evoked potential; the system generates a command using the evoked brain signal when the user gazes at a target stimulus. A typical ERP system does not consider the possibility that a user is gazing at a stimulus without any intention of control. However, this situation would commonly occur in the real-world environment. For instance, a user may require some time to rest for a variety of reasons (boredom, tiredness [51], eye-fatigue [52], etc [53].), or they may want to perform some other mental task while using the ERP system. In our study, the passive gazing task achieved 81.4% decoding accuracy (see Fig 2). The passive gazing task in our study was not defined as a non-intentional mental state; nevertheless, it suggested that a conventional ERP system could generate an unintentional command due to the characteristic of exogenous evoked potentials [54]. To block the unintentional command, one possible method is to not gaze at any visual-stimuli or to ignore the target stimuli so that the classifier output does not exceed the pre-defined threshold. The majority of ERP systems do not consider such situations; furthermore, these physical actions to avoid unintentional commands are not natural behaviors in the human-computer interface system. For disabled users, particularly those who have a neuromuscular disease, it would be difficult to avoid gazing at the target and non-target stimuli by exercising some degree of motor control.

The two-stage BCI system is an idea wherein the target symbol is estimated using a target decoder, and these target symbols are selected by the active decoder if the user remains in the intentional mental state. Thus, it increases system usability by preventing unintentional commands.

Furthermore, active detection through a user voluntary task could also be used in developing the newer BCI applications such as binary decision, asynchronous, or auditory BCI systems. Binary decision systems (i.e., yes or no) have been developed for the final stages of disease and mostly use a selective attention paradigm for the stimulus [55]. Halder et al. [24] proposed an auditory BCI system using three external auditory stimuli with different directions and sounds. The subjects selectively attended to one of the sounds to generate a command. Asynchronous BCI control [56] is also important issue in real-life settings, and it determines a target as not only the desired command but also when the user actually wants to input that command. In all of these avenues of newer BCI applications, our technique of active detection could lead to more reliable interactions.

With the application of an active attention paradigm, usability and user convenience can be greatly enhanced. The distinction between active and passive mental states allows for a constant presentation of minimally imposing stimuli that provide access to a needed command whenever purposefully generated by the user while at the same time not necessitating large, distracting, robust visual presentations. For example, a user could call a nurse or activate any other system at their own discretion without the fatiguing, distracting encumbrances typically employed in BCI. This method leads to the possible development of a much less stimulus-dependent BCI system that could be applied in a greater number of environments. By virtue of the user voluntary mental task, visual stimuli can be minimized and then embedded into various mobile devices, such as a smartphone or wheelchair, thus allowing greater portability and less intrusion into any environment in which the user will have a need for the BCI.

## Conclusion

In this study, we examined the ERP response and its decoding accuracy with the hypothesis that different exogenous potentials are evoked according to the level of effort required for the mental task. Three mental states were defined: passive attention (i.e., passively gazing) and active attention (i.e., counting and pitch-imagery tasks). We proposed a novel mental task, i.e. pitch-imagery, and it was considered to be the highest level of user mental effort for this experiment.

First, our experiment results indicated that the active attention tasks generated larger P300 and N500 components, which significantly influenced the decoding accuracy for target and non-target trials. Second, we found that the N500 component is highly dependent on the task; pitch-imagery, which required the highest level of mental effort, elicited a stronger N500 deflection than the counting or passive gazing tasks. The pitch-imagery task also achieved significantly improved decoding accuracy in the early stage of the sequential procedure (see Fig 2), and showed relatively small attenuation of N500 component between the sessions (see Fig 9). Third, we demonstrated the possibility of classifying the user’s mental state as either passive or active for more general use in other ERP-based BCI techniques.

The counting and pitch-imagery tasks are actively generated by the user, and performance highly depends on the user’s ability. In other words, system performance is mainly affected by user-side factors rather than the experimental stimuli parameters. This will benefit the development of user-friendly BCI systems as the system does not require a specific type of visual stimulus (e.g., human face, color, shape and size).

However, evoked potentials from the active tasks result from the different brain processes associated with the specific task, and the brain patterns and performance are highly subject-dependent. Although the pitch-imagery task showed more than 5% improved performance than the counting task (see Fig 2), we are not suggesting that the pitch-imagery task is the optimal user voluntary mental task. Therefore, the proper task in any ERP-based BCI system should comprehensively consider many factors such as type of application, user-specific capability, preference of the tasks, etc.

In our future work, we will focus on testing (and possibly adapting) this system with the help of a clinical subject population. We hope to bring this technology from the test bench to the clinical subjects for whom the design was intended. Our dataset is available on figshare (https://dx.doi.org/10.6084/m9.figshare.8091242).
